# Personalized Diets based on the Gut Microbiome as a Target for Health Maintenance: from Current Evidence to Future Possibilities

**DOI:** 10.4014/jmb.2209.09050

**Published:** 2022-10-25

**Authors:** Eun-Ji Song, Ji-Hee Shin

**Affiliations:** Research Group of Personalized Diet, Korea Food Research Institute, Jeollabuk-do 55365, Republic of Korea

**Keywords:** Diet, personalized nutrition, gut microbiota, enterotype, human health

## Abstract

Recently, the concept of personalized nutrition has been developed, which states that food components do not always lead to the same metabolic responses, but vary from person to person. Although this concept has been studied based on individual genetic backgrounds, researchers have recently explored its potential role in the gut microbiome. The gut microbiota physiologically communicates with humans by forming a bidirectional relationship with the micronutrients, macronutrients, and phytochemicals consumed by the host. Furthermore, the gut microbiota can vary from person to person and can be easily shifted by diet. Therefore, several recent studies have reported the application of personalized nutrition to intestinal microflora. This review provides an overview of the interaction of diet with the gut microbiome and the latest evidence in understanding the inter-individual differences in dietary responsiveness according to individual baseline gut microbiota and microbiome-associated dietary intervention in diseases. The diversity of the gut microbiota and the presence of specific microorganisms can be attributed to physiological differences following dietary intervention. The difference in individual responsiveness based on the gut microbiota has the potential to become an important research approach for personalized nutrition and health management, although further well-designed large-scale studies are warranted.

## Introduction

Microbes inhabit various parts of the human body including the skin, urogenital tract, oral cavity, and gastrointestinal (GI) tract; approximately 95% of the microbes live in the GI tract. [1]. The gut microbiota increases the bioavailability of food ingredients, produces metabolites that are transported to different parts of the body, and is involved in the metabolic transformation of food ingredients. [2]. Maintenance of gut microbiota homeostasis is closely related to human health, and an imbalance is observed in several diseases such as obesity, inflammatory bowel disease, and non-alcoholic fatty liver disease [3]. Intrinsic factors such as gender, ethnicity, and age [4, 5], and extrinsic factors such as diet, hygiene, antibiotic use, and mode of delivery [6] modulate the composition of the gut microbiome. Most of all, diet is a major environmental factor that shapes gut microbial communities [7].

With the success of the Human Genome Project in 2003, the term “Nutrigenomics” was coined in the field of nutritional sciences. Nutrigenomics is a study area that focuses on the relationship between diet and genetics and how their interactions provide positive and negative effects on human health [8]. As is the basis of this study, inter-individual variation in gene sequences can influence the bioavailability and metabolism of specific nutrients; this concept has provided a scientific basis for personalized nutrition. Numerous studies have shown that the bioavailability of specific dietary components differs from person to person [9-11]. In the same manner as personalized medicine, personalized nutrition strategies aim to design tailored dietary recommendations that vary interpersonally, avoiding a “one-size-fits-all” approach. Recent research has shown that intestinal microbes can provide new opportunities for personalized nutrition [12, 13]. Compared to the genome, where immediate and dramatic changes in the environment are rigid, the human gut microbiota fluctuates more owing to the influence of various environmental factors. Diet is an essential factor in this variability; the bidirectional relationship between diet and gut microbiota is closely maintained and it influences the host’s health status [7]. Furthermore, since individual eating patterns and gut microbiota are relatively diverse, the bidirectional relationship between diet and gut microbiota can contribute to inter-individual variability in physiological responses. In this context, there has been growing interest in the potential of personalized nutrition based on gut microbiota to manage human health.

Here, we summarize the interactions between dietary factors and gut microbiota in consideration of the individual baseline gut microbiota. Furthermore, we discuss the current status of research on personalized nutrition based on gut microbiota in people with various diseases. This review will help us to understand the potential of gut microbiome-based personalized diets.

## Dietary Macro- and Micronutrients and Their Interactions with the Gut Microbiome

### Macronutrients

Carbohydrates are the primary energy source, supplying approximately 50% of our total daily energy needs [14]. Stephen *et al*. [15] estimated that approximately 2–20% of unabsorbed dietary carbohydrates enter the colon. Simple carbohydrates from the diet are completely absorbed in the small intestine, whereas complex carbohydrates, such as resistant starch, non-starch polysaccharides, and oligosaccharides, are not absorbed in the small intestine and thus reach the colon. Therefore, the term “microbiota-accessible carbohydrates (MACs)” has been proposed to define these complex carbohydrates that cannot be utilized by the digestive system of the host, but are metabolically available to the gut microbiota due to their specific enzymes, such as glycoside hydrolases and polysaccharide lyases [16]. MACs are typically referred to as dietary fiber. Accumulating data suggest that dietary fibers have direct beneficial effects on human health via alterations in gut microbiota composition and diversification of the gut microbiota. Moreover, the primary end-products of dietary fiber from gut microbiota fermentation are short-chain fatty acids (SCFAs), which play an important role in human health. Acetate, butyrate, and propionate, the three major SCFAs, are energy sources for colonocytes. In addition to being the energy source of colon cells, SCFAs can regulate host gene expression by inhibiting histone deacetylases (HDACs), and can also regulate energy metabolism, intestinal homeostasis, and immune response by binding several G protein-coupled receptors (GPCRs) [17]. Numerous studies have demonstrated that SCFAs can protect against inflammation, the infiltration of pathogenic bacteria, carcinogenesis, and diet-induced obesity [18].

Dietary proteins also influence the alteration of gut microbiota composition and function, and these changes are associated with host physiology [19]. Approximately 10% of ingested proteins enter the colon [20] and can serve as a substrate for colonic microbiota. The nature of residual proteins fermented by gut microbiota includes SCFAs, gases, and nitrogen compounds, and the nature of metabolites is determined by the amount and content of amino acids in the source of the dietary protein [21]. L-carnitine, which is specifically abundant in red meat, is converted into trimethylamine by the gut microbiota and is consequently transformed into trimethylamine oxide (TMAO) in the liver. Elevated circulating levels of TMAO have been linked to the promotion of atherosclerotic lesions and have been discovered to be a risk factor for cardiovascular disease [19]. Moreover, high protein consumption leads to the increased generation (via gut microbial fermentation) of genotoxic secondary metabolites, including phenols, ammonia, and polyamines, which have been associated with gastrointestinal cancer. In contrast, some metabolites produced by the bacterial fermentation of tryptophan, including indole-3-acetic acid and indolepropionic acid, have been shown to attenuate inflammation [22] and maintain intestinal mucosal homeostasis [23].

Dietary fat is mostly utilized and absorbed in the small intestine, but a small percentage of fat also reaches the colon [24]. The gut microbiota plays an important role in cholesterol metabolism and lipid digestion through the regulation of bile acid homeostasis. Primary bile acids are synthesized from dietary cholesterol, conjugated with taurine or glycine in the liver, and released into the duodenum for dietary lipid digestion. In the colon, primary bile acids are biotransformed into secondary bile acids by gut microbial deconjugation enzymes. These, in turn, are reabsorbed from the gut back into the liver and alter the composition of the circulating bile acid pool [19]. Changes in the composition of the bile acid pool affect hepatic bile acid synthesis via farnesoid X receptor activity, which regulates dynamic signaling molecules [25]. Another mechanism proposed to explain the link between gut microbiota and dietary fat is through lipopolysaccharides (LPSs), which are part of the cell wall of gram-negative bacteria. A high-fat diet, particularly saturated fat, increases LPS-expressing bacteria and gut permeability, leading to elevated levels of circulating LPSs. Circulating LPSs induce a potent inflammatory state through the Toll-like receptor 4 (TLR4) signaling pathway [17], which is involved in the development of metabolic diseases, such as obesity, insulin resistance, and cardiovascular disease.

### Micronutrients and Phytochemicals

The human gut microbiota synthesizes various B vitamins, as well as vitamin K. Microbial-derived vitamins are absorbed by colonocytes and are involved in the physiological properties of the host, such as energy metabolism, indicating that the gut microbiota contributes to systemic vitamin status. Moreover, a series of studies have shown that the composition and function of the gut microbiota are affected by micronutrient status [26]. In addition to vitamins, minerals can also alter the gut microbiota. For example, iron is essential for pathogen growth; therefore, pathogens compete with commensal gut microbiota for iron. In addition to micronutrients, dietary polyphenols, which are abundant in various fruits and vegetables, are mostly metabolized by gut microbial enzymes owing to their poor bioavailability for host enzymes [27]. There have been reports of the differential bioconversion ability of various phytochemicals depending on an individual’s intestinal microbiome [28]. Gross *et al*. [29] examined the biotransformation capacity of polyphenols in black tea and red wine juices of microbes in the stool of healthy adults and found that the profile and time course of polyphenol metabolites varied among individuals. Similar to these results, the bioconversion of epigallocatechin-3-O-gallate contained in green tea and epicatechin mainly contained in plant-based food also showed that there was a difference depending on the individual intestinal microbes [30, 31]. Polyphenols can also affect the composition of the gut microbiota. For example, recent studies have shown that polyphenols induce a significant increase in the proportion of beneficial bacteria, such as *Bifidobacterium* [32] and *Lactobacillus* spp., but decrease the proportion of *Clostridium* spp. [33]. Taken together, a variety of food components and the gut microbiota have a two-way relationship. Nevertheless, because individuals do not consume single foods or nutrients in isolation, it is crucial to ascertain the specific effects of dietary patterns on gut microbiota composition and human health [34]. It should also be considered that the metabolism or activity of food ingredients may differ depending on the composition of individual gut microbes.

## Inter-Individual Differences in Dietary Responsiveness according to Individual Baseline Gut Microbiota

### Differential Response to Diet according to Gut Microbiota Diversity

The diversity and composition of human gut microbiota vary considerably among individuals depending on intrinsic or extrinsic factors, such as diet, hygiene, antibiotic usage, and mode of delivery [35]. Among the factors, diet plays a crucial role in gut microbial composition, and its effects prevail over genetic influences [36, 37]. The first three years of life have a great impact on the development of the gut microbiota, and gut microbial diversity increases with age until it becomes stable in adulthood [38]. Adults tend to establish habitual eating patterns and are less prone to trying new food types [39]. The established diversity of the gut microbiota also influences the variability of individual dietary responsiveness. Several recent studies have demonstrated that low microbial diversity contributes to the instability of individual gut microbiota following perturbations, such as dietary intervention [40, 41] (Table 1). Changes in gut microbes or phenotypes after a specific intervention by baseline diversity are predicted to be related to the resilience of individual gut microbes [42]. Generally, the adult gut microbiota composition can maintain its stable state; however, its equilibrium can be disrupted by external disturbances, after which it recovers its stable state. In the context of resilience, a high bacterial diversity at baseline indicates a low level of dietary responsiveness because it maintains its stable state, whereas a lower initial microbiota diversity seems to be favorable to alteration accordingly. In addition to stability, phenotypic changes due to the baseline gut microbial diversity have also been reported in several papers. A study conducted on overweight adults revealed that individuals with a low microbial gene count showed less improvement in systemic inflammation and risk of dysmetabolism than those with a high microbial gene content after an energy-restricted diet intervention [43] (Table 1). Additionally, another study in overweight individuals showed that a higher count of total bacteria (richness) resulted in greater changes in body weight loss after a calorie-restricted diet [44] (Table 1). High phenotypic responsiveness is not always helpful. In the case of trimethylamine-N-oxide (TMAO), a compound that is metabolized by gut microbiota and associated with an increased risk of cardiovascular disease, a low microbial diversity has been reported to be associated with a greater response to TMAO (increased TMAO production) [45] (Table 1).

The presence of specific microorganisms can be attributed to physiological differences after dietary intervention. For instance, Korem *et al*. [46] reported that two types of bread (whole-grain bread vs. refined wheat bread) did not show a difference in glycemic response or dramatic changes in the intestinal microflora (Table 1). Instead, they found that the response of blood glucose to each bread type was different for individuals, and the baseline abundance of *Coprobacter fastidiosus* and *Lachnospiraceae bacterium* *3_1_46FAA* can predict the glycemic response to different bread types in different individuals. Choline, an essential nutrient and methyl donor, has the potential to contribute to fatty liver disease, and one study has shown that the relative abundance of *Gammaproteobacteria* and *Erysipelotrichia* at baseline lowers the liver fat to spleen fat ratio after a choline-depletion diet [47] (Table 1). In the case of prebiotic intervention, a lower relative abundance of *Bifidobacterium* at baseline was associated with an increase in prebiotic inulin supplementation in healthy adults [48] (Table 1). Responders were those with alleviation of inflammatory bowel disease (IBS)-symptom severity following low-FODMAP diet intervention. Their baseline taxonomic characteristics exhibited higher relative abundances of *Phascolarctobacterium* [49], *Bacteroides*, *Ruminococcaceae*, and *Faecalibacterium parusnitzii* [50] (Table 1). Furthermore, overweight participants with lower abundances of *Lactobacillus*, *Leuconostoc*, and *Pediococcus* at baseline showed less weight loss and rapidly regained weight after an energy-restricted diet and normal diet, respectively. Using the same participants from Kong *et al*. [51], Dao *et al*. [52] showed that the basal abundance of *Akkermansia muciniphila* was inversely correlated with improvements in insulin sensitivity and lipid metabolism (Table 1). Furthermore, a higher abundance of *A. muciniphila* at baseline led to greater body fat loss after energy-restricted diet intervention. Additionally, Jie *et al*. [53] reported that *Blautia wexlerae* and *Bacteroides dorei* can predict weight loss in a calorie-restricted diet (Table 1). These studies raise the possibility that the efficacy of inter-individual variation in response is closely related to an individual’s intrinsic microbial community and its features.

### Differential Response to Diet according to Enterotypes

Considering the differences in the gut microbiota of all individuals, it is difficult to study their response to diet. An efficient approach could be to stratify individuals appropriately based on their gut microbiota composition. Stratification of bacterial communities based on differences in the enrichment of microbial taxa can simplify the complexity of gut microbiota. Arumugam *et al*. [54] first identified three bacterial groups in humans that are represented by different proportions of dominant bacterial clusters: *Bacteroides* (enterotype 1), *Prevotella* (enterotype 2), and *Ruminococcus* (enterotype 3). An intra-continental study showed that two (*Bacteroides* and *Ruminococcaceae*), four (*Faecalibacterium*, *Bacteroides*, *Prevotella*, and *Clostridiales*), and two (*Prevotella* and *Bacteroides*/*Bifidobacterium*) enterotypes were identified in the American, European, and Asian continents, respectively [55]. Gut microbiota co-evolve by reflecting the region where the host lives and their dietary history [56]. A cross-sectional analysis of dietary information and the gut microbiome showed that the *Prevotella*-dominated enterotype in humans is associated with a high intake of fiber, carbohydrates, and simple sugars, whereas a *Bacteroides*-dominated enterotype in humans is associated with a high intake of animal fat and protein [57]. Differences in functional abilities depending on enterotype-specific microorganisms can affect human metabolism functions, such as energy homeostasis and appetite control [58]. *Bacteroides* and *Prevotella*, which are representative enterotype microorganisms, have different metabolic abilities for carbohydrates, proteins, fats, fibers, minerals, and vitamins [59, 60]. In particular, a higher fiber utilization capacity was observed in the *Prevotella*-dominated enterotype. A study on dietary fiber supplementation, including fructooligosaccharides, sorghum bran, and corn arabinoxylan, showed that total SCFA and propionate levels were significantly higher in individuals with the *Prevotella*-dominated enterotype than in those with the *Bacteroides*-dominated enterotype [61]. Moreover, batch culture fermentation of isomaltooligosaccharides with human feces resulted in higher propionate and butyrate levels with the *Prevotella*-dominated enterotype than with other enterotypes [62]. In contrast, the *Bacteroides*-dominated enterotype was more proficient in degrading and utilizing polyguluronate and polymannuronate than the *Prevotella*-dominated enterotype, resulting in higher amounts of total SCFAs and butyrate [63]. Recently, metagenome analysis found that enterotypes showed clear genetic differentiation in terms of their functional catalog of genes, especially for genes involved in saccharolytic, proteolytic, and lipolytic profiles [64, 65]. Differences in the composition of the gut microbiota and their genetic composition are expected to affect the host’s dietary breakdown/use and health.

Several recent studies have found evidence that enterotypes may be useful for predicting responses to diets. The *Prevotella*-to-*Bacteroides* ratio has been found to be closely related to alterations in body fat [66, 67], weight [67, 68], BMI [69], total cholesterol [70], and hormonal responses [71, 72] according to dietary intervention (Table 1). A calorie-deficit diet led to greater BMI and weight loss in individuals with a higher *Prevotella*-to-*Bacteroides* ratio [67, 69] (Table 1). A whole grain wheat diet led to greater body weight loss in individuals with the *Prevotella*-dominated enterotype compared to the refined wheat diet, whereas individuals with the *Prevotella*-defected enterotype showed no differences in body weight [68]. Fiber-rich bread led to an improvement in glucose and insulin levels in individuals with a high *Prevotella*-to-*Bacteroides* ratio [71] (Table 1). Administration of the probiotic GP-2 improved obesity-related markers, such as waist circumference, total fat area, and visceral fat [73]. The decrease in obesity-related markers was greater in the *Prevotella*-dominated enterotype group. The effective responses related to body fat, weight loss, and BMI were focused on the *Prevotella*-dominated enterotype. However, results showing a high response to *Bacteroides*-dominated enterotypes have also been reported. A study on capsaicin intervention showed that butyrate levels were significantly higher in the *Bacteroides*-dominated enterotype following dietary capsaicin intake [72] (Table 1). When consuming capsaicin, the beneficial effects on gastrointestinal hormones (*i.e.*, GLP-1, GIP, and ghrelin) were associated with a higher abundance of *Bacteroides*. Interestingly, Hjorth *et al*. [66] reported that individuals with high *Prevotella* lost more body weight after the New Nordic Diet with high fiber than after an average Danish diet with lower fiber, whereas individuals with low *Prevotella* showed no differences in body weight (Table 1). In contrast, lower total plasma cholesterol was observed in the low *Prevotella*-to-*Bacteroides* ratio group than in the high *Prevotella*-to-*Bacteroides* ratio group after intervention with the New Nordic Diet [70] (Table 1). Despite the limited number of studies, it can be inferred that the phenotypic benefits obtained from the same diet are different for each enterotype.

There are some controversies surrounding enterotypes, with some disagreement about whether they are just methodological artifacts, truly discreet clusters, or just gradients; in addition, their true relevance in microbial community dynamics and host health is still debated [74-76]. However, it is worth exploring because reports on differential dietary responses are accumulating. In order to find answers to the unanswered questions and to assess the usefulness of enterotypes, large scale studies are necessary. In addition, research on the differences in the microbial composition of enterotypes, and the comparison of microbial functions through metagenomic analysis or comparative genome study, is warranted. In addition, understanding the mechanism and the cause for the differential response of each enterotype is essential. Future studies could present enterotypes as an advanced tool in personalized nutrition research.

## Microbiome-Associated Dietary Intervention in Diseases

### Dietary Intervention in Irritable Bowel Syndrome (IBS)

According to the personalized nutrition paradigm, the effect of the same diet does not result in the same responsiveness in all patients. The gastrointestinal tract is an organ where most microorganisms in the human body are clustered, and it is also a site where the immune response of intestinal microorganisms and the host occurs. Various gastrointestinal and immune-related diseases are considered to be related to the gut microbiota [77]. In addition to the gut microbiome, dietary factors are also important components in the management of gastrointestinal health and disease. Thus, certain nutritional therapies have been designed to help relieve symptoms in people with gastrointestinal disorders, such as a low-fermentable oligosaccharides, disaccharides, monosaccharides, and polyols (FODMAP) diet for people with irritable bowel syndrome (IBS). The low-FODMAP diet reduced the consumption of slowly absorbed or indigestible carbohydrates, thereby reducing IBS symptoms [78]. More recently, differences in the effects of specific diets according to the baseline gut microbiota have been observed in participants with gastrointestinal diseases. For example, studies have reported that patients with IBS have different responsiveness depending on the baseline status of specific gut microbiota after consuming a low-FODMAP diet [49, 50]. Higher abundances of *Phascolarctobacterium*, *Bacteroides*, *Ruminococcaceae*, and *Faecalibacterium prausnitzii* were associated with the alleviation of IBS symptom severity after low-FODMAP diet intervention in individuals with IBS [49, 50]. These bacterial taxa may be capable of fermenting nondigestible carbohydrates. Chumpitazi *et al*. [50] suggested that the difference in the glycolytic ability of intestinal microbes could be a biomarker that predicts the responsiveness of a low-FODMAP diet. Similarly, Vervier *et al*. [79] showed that responders with improved clinical responsiveness to a low-FODMAP diet in IBS patients had gut microbiota rich in genes involved in carbohydrate metabolism. However, large-scale randomized controlled studies of responsiveness to specific dietary interventions based on gut microbiota are still lacking, and the exclusion of factors (*e.g.*, genetic and environmental factors) that may affect an individual’s responsiveness to a diet should be sufficiently considered.

### Dietary Intervention in Neuropsychiatric Disorders

In terms of neuropsychiatric disorders, several studies have shown the potential of gut microbiome-based personalization. A study of Zhang *et al*. [80] showed that the efficacy of nutritional treatment for epilepsy depends on the gut microbiota. The ketogenic diet is a high-fat, low-carbohydrate diet designed to increase the levels of ketone bodies and is an effective nutritional treatment option for epilepsy that relieves seizure symptoms [80]. After 6 months of ketogenic diet treatment, responsive patients with ameliorated seizing had significantly lower relative abundances of *Ruminococcaceae*, *Lachnospiraceae*, *Rikenellaceae*, *Clostridiales*, *Clostridia*, and *Alistipes* than did non-responsive patients [80]. In the case of autism, there is no study showing a difference in the response to a specific diet depending on the gut microbiome, but the results of some studies suggest that the relationship between diet and the gut microbiome may influence the disorder. Berding *et al*. [81] reported that the eating patterns of people with autism were divided into two distinct patterns according to the intake of vegetables, legumes, nuts, seeds, and starchy vegetables, and that each eating pattern was associated with different specific bacteria. This trend may be related to the eating behavior of patients with autism [81-83], and the change in the gut microbiota related to this eating behavior shows potential for application in autism management and interventional treatment.

Preventing disease, as well as treating or managing it, will help people manage their health through the gut microbiome. Whereas previous studies focused on the difference between the gut microbes of responders and non-responders through intervention studies, recent studies that attempted to predict the response to diet through actual gut microbes using a machine learning approach have been reported. Machine learning approaches aim to integrate and learn various patterns from datasets and discover predictive algorithms that enable the discovery of new biomarkers [84]. Zeevi *et al*. [13] demonstrated that unique individual gut microbial profiles can predict postprandial blood glucose levels owing to varying responses to the same foods in individuals. Similarly, Korem *et al*. [46] reported that the glycemic response to two types of bread (whole-grain bread vs. refined wheat bread) can be predicted based on the baseline abundance of specific bacterial taxa. Studies of microbiome-based personalized dietary responses through machine learning are still relatively rare and present challenges. Because the quality and quantity of input data are important for machine learning approaches [85], well-validated information on the host–microbiome–diet interaction is required to provide a wide range of gut microbiota-based personalized nutrition solutions.

## Conclusion

Precision nutrition aims to develop nutritional recommendations based on parameters that change and interact with each individual’s internal and external environments. Precision nutrition might be essential to prevent the development of certain diseases and maintain the health status of a normal individual through guidance on preferred diet or food, based on the various host parameters, in near future. To increase the efficiency of precision nutrition, it is important to secure information about the individual’s gut microbiome and their reactivity to each diet or food item. In this review, we evaluated the changes in the dietary components according to the individual gut microbiome, the differences in reactivity according to gut microbial enterotypes, and the dietary interventions for improving the symptoms of various diseases. In particular, attempts have been made to predict the effect of diet based on information on the gut microbiota using machine learning in metabolic diseases. However, applying this new approach to a wide range of individuals will require various large-scale and well-designed clinical trial results for the responsiveness to diet based on gut microbiota. In addition, follow-up observations will be needed to determine whether personalized nutrition based on gut microbiota is sustainable and has a more positive effect on clinical outcomes than do conventional nutritional approaches.

## Figures and Tables

**Table 1:** Characteristics of nutrition intervention studies on differences in phenotypes based on individuals’ gut microbiota.

Variable factors	Nutritional Intervention or challenge	Duration	Related disease	Study design	Participants (n)	Major conclusion	Citation
Baseline enterotype	New Nordic Diet (high in fiber and whole grain) vs. average Danish diet	26 weeks	Metabolic syndrome	Randomized controlled diet intervention	Participants with increased waist circumference (*n* = 62)	High P/B ratio: greater body fat loss	[66]
	New Nordic Diet (high in fiber and whole grain) vs. average Danish diet	26 weeks	Metabolic syndrome	Randomized controlled diet intervention	Participants with central obesity and components of metabolic syndrome (*n* = 62)	Low P/B ratio: greater decreased total cholesterol	[70]
	Barley kernel-based bread vs. White wheat flour bread	3 d	NA	Randomized cross-over diet intervention	Healthy adults (*n* = 39)	High P/B ratio: improvement in glucose and insulin responses	[71]
	500 kcal/d energy deficit diet	24 weeks	Metabolic syndrome	Randomized, controlled, parallel design	Participants with overweight (*n* = 52)	High P/B ratio: increased weight loss and body fat loss	[67]
	Calorie restriction diet (approximately 40% energy deficit)	3 weeks	NA	Uncontrolled longitudinal study	Non-obese adults (*n* = 41)	*Prevotella* enterotype: increased BMI loss	[69]
	Low-capsaicin vs. high-capsaicin	6 weeks	NA	Controlled cross-over diet intervention	Healthy adults (*n* = 12)	*Bacteroides* enterotype: increased glucagonlike peptide 1, gastric inhibitory polypeptide, and decreased ghrelin	[72]
Baseline microbial diversity	Weight maintenance diet vs. standard diets supplemented with resistant starch vs. standard diets supplemented with non-starch polysaccharides vs. weight-loss diet	10 weeks	Metabolic syndrome	Randomized cross-over diet intervention	Obese adult males (*n* = 14)	Low baseline diversity: more unstable gut microbial change after dietary intervention	[40]
	10 g vs. 40 g of dietary fiber	5 d	NA	Randomized cross-over diet intervention	Healthy adults (*n* = 19)	Low baseline microbiota richness: more unstable gut microbial change	[41]
	Energy-restricted high-protein diet vs. weightmaintenance diet	6 weeks	Metabolic syndrome	Randomized cross-over diet intervention	Overweight and obese adults (*n* = 49)	Low baseline bacterial gene count: less improvement in risk of dysmetabolism and inflammation	[43]
	Calorie restriction diet (approximately 10–40% energy deficit) and increased physical activity	10 weeks	Metabolic syndrome	Standardized diet advice provided	Overweight adolescents (*n* = 36)	High baseline bacterial richness, *Bacteroides fragilis*, *Clostridium leptum*, and *Bifidobacterium catenulatum*: increased body weight loss	[44]
	TMAO-rich diet vs. choline-rich diet vs. carnitinerich diet vs. control diet	1 meal	NA	Randomized controlled cross-over diet intervention	Healthy adult males (*n* = 40)	Lower baseline bacterial diversity, higher Firmicutes/ Bacteroidetes ratio and abundance of *Clostridiales*: high TMAO production	[45]
Baseline specific gut microbial taxa	Sourdough wholegrain bread vs. white wheat bread	1 week	NA	Randomized cross-over trial	Healthy adults (*n* = 20)	Relative abundance of *Coprobacter fastidiosus* and *Lachnospiraceae bacterium* 3_1_46FAA can predict glycemic response to different bread types	[46]
	Placebo (maltodextrin, 8 g/ day) vs. inulin (5 g/ day and 8 g/day)	2 weeks	NA	A doubleblind, placebocontrolled, crossover study	Healthy adults (*n* = 30)	Lower abundance of *Bifidobacterium*: higher increase in *Bifidobacterium* after inulin supplement	[48]
	Energy-restricted, high-protein diet vs. weight maintenance diet	6 weeks	Metabolic syndrome	Randomized cross-over diet intervention	Obese and overweight adults (*n* = 50)	Lower abundance of *Lactobacillus*/ *Leuconostoc*/ *Pediococcus*: higher plasma insulin, IL-6, adipose tissue inflammation, and less weight loss and rapidly regained weight during the stabilization period	[51]
	Energy-restricted, high-protein diet vs. weight maintenance diet	6 weeks	Metabolic syndrome	Randomized cross-over diet intervention	Obese and overweight adults (*n* = 49)	Higher abundance of *Akkermansia muciniphila*: higher improvement in insulin sensitivity, lipid metabolism, and greater body fat loss	[52]
	Calorie restriction (30–50% energy deficit)	6 months	Metabolic syndrome	Standardized diet advice provided	Overweight adults (*n* = 83)	Relative abundance of *Blautia wexlerae* and *Bacteroides dorei* can predict weight loss	[53]
	Low-FODMAP diet vs. Traditional IBS diet	4 weeks	Gastrointestinal disease	Randomized controlled diet intervention	Adults with IBS (*n* = 61)	Higher abundance of *Phascolarctobacterium*: lower IBS-symptom severity score (IBS-SSS) after low-FODMAP diet intervention	[49]
	Low-FODMAP diet vs. typical TACD	2 d	Gastrointestinal disease	Randomized controlled cross-over diet intervention	Children with IBS (*n* = 33)	Higher abundance of *Bacteroides*, *Ruminococcaceae*, *Faecalibacterium prausnitzii*: less daily abdominal pain	[50]
	Conventional diet (550 mg/70 kg body weight) vs. choline-depletion diet (<50 mg/70 kg body weight) vs. choline-repletion diet (850 mg/70 kg body weight)	10 d (conventi onal diet), 42 d (depletion diet), 10 d (repletion diet)	NA	Parallel standardized diet	Healthy female adults (*n* = 15)	Higher abundance of *Gammaproteobacteria* and *Erysipelotrichia* at baseline: lower liver fat to spleen fat ratio after choline-depletion diet	[47]

NA = not applicable; FODMAP= fermentable oligosaccharides, disaccharides, monosaccharides, and polyols; P/B = *Prevotella*/*Bacteroides*; IBS: irritable bowel syndrome; TMAO: trimethylamine‐N‐oxide; TACD: typical American childhood diet.
